# Gender-responsive infectious disease models: Guidelines and systematic scoping review

**DOI:** 10.1016/j.dialog.2026.100329

**Published:** 2026-07-22

**Authors:** Alisa Hamilton, Indira Prihartono, Yamato Okura, Naomi Rankin, Samee Saiyed, Jeremy Ratcliff, Ayo Oladimeji, Tak Igusa, Lauren Gardner, Rosemary Morgan

**Affiliations:** a Whiting School of Engineering, Johns Hopkins University, USA; b Bloomberg School of Public Health, Johns Hopkins University, USA; c Johns Hopkins University Applied Physics Laboratory, USA

**Keywords:** Infectious disease modeling, Mathematical modeling, Gender disparities, Health equity, HIV, COVID-19, Malaria

## Abstract

**Background:**

Sex and gender influence infectious disease outcomes through biological and social mechanisms. Gender norms, roles, and behaviors affect exposure, care-seeking, and service access, yet many infectious disease models overlook these factors.

**Methods:**

To provide guidance to researchers considering sex/gender in epidemiological models, we propose structured guidelines for developing gender-responsive infectious disease models, adapted from an established monitoring and evaluation framework. Recommendations include: 1) using sex/gender-disaggregated or sex/gender-specific populations; 2) incorporating an additional social stratifier (e.g., age, race/ethnicity); 3) maintaining stratification in results; 4) addressing the needs, rights, and preferences of gender groups; and 5) grounding models in context with participation from target groups. To provide examples, we conducted a scoping review using these criteria, drawing from HIV, COVID-19, and malaria literature.

**Findings:**

Of 7303 studies screened, 45 met inclusion criteria. Most modeled HIV (96%), used compartmental (73%) or agent-based (22%) approaches, and included sexual orientation or occupation as an additional stratifier. Studies contextualized their models by discussing local healthcare systems, unequal access to care, stigma, gender norms in partnership dynamics, and occupational risk.

**Interpretation:**

Gender-responsive modeling is feasible but uncommon beyond sexually transmitted infections. Applying intersectional, context-specific approaches can reveal disparities and inform equitable, efficient interventions. These guidelines can be adapted to address other health disparities.

## Highlights

• Sex and gender impact infectious disease transmission.
• Gender considerations often not incorporated in model and scenario design.
• Gender-responsive models go beyond sex/gender disaggregation.
• Gender dimensions and active approaches make models more gender-responsive.
• Examples from HIV can inform gender-responsive models of other diseases.

## 1 Introduction

Sex and gender shape both the biological and social dimensions of infectious disease dynamics. Sex, defined as the biological classification of individuals based on physical and physiological attributes, such as chromosomes, reproductive anatomies, and hormonal profiles, is typically categorized as female and male, although variations exist [1]. Sex is an important variable that can influence susceptibility and outcomes due to genetic, hormonal, and immunological variations [2], [3], [4]. For example, studies indicate that female coronavirus disease (COVID-19) patients exhibited stronger antibody responses, whereas male COVID-19 patients were more likely to experience higher severity and mortality [5], [6]. Anatomical differences related to sex play an important role in human immunodeficiency virus (HIV) transmission, contributing to markedly higher infection risk among transgender women (66-fold) and cisgender women engaging in receptive penile-vaginal intercourse (2-fold) compared with men [7], [8].

Gender refers to social norms and roles attributed to being a woman, man, or gender minority individual. These dynamics influence infection risk and disease progression through differences in occupational exposure, mobility, social roles, and health-seeking behaviors [9]. For example, women's household-centered activities can increase exposure to *Aedes* mosquitos in dengue, chikungunya, and Zika [10], [11], while men's work-related mobility, as seen in Southeast Bangladesh, can increase their malaria risk and facilitate transmission in non-endemic areas [12].

Researchers increasingly recognize sex and gender as important aspects in infectious disease dynamics [13], [14], [15], [16], yet practical guidance for incorporating these measures into disease dynamic models remains limited. This study aims to address this gap by providing structured guidelines and examples for designing gender-responsive infectious disease models, following the gender-responsive monitoring and evaluation (M&E) framework developed by Monitoring and Action for Gender Equity (MAGE) [17]. While existing guidelines focus on stratifying models by sex or other demographic groups [14], [16], our approach goes further by outlining how to apply an intersectional lens, address the needs, rights, and preferences of different gender groups, and contextualize models to increase decision making relevance. We conduct a systematic scoping review to identify examples of gender-responsive models using the MAGE definition from three commonly modeled diseases with distinct transmission routes (HIV, COVID-19, and malaria). By using a gender-responsive approach, infectious disease models can better inform decisions to improve efficient resource use, reduce inequities, and improve overall population health.

## 2 Applying a gender-responsive M&E framework to guide gender-responsive infectious disease modeling

**Fig. 1:**
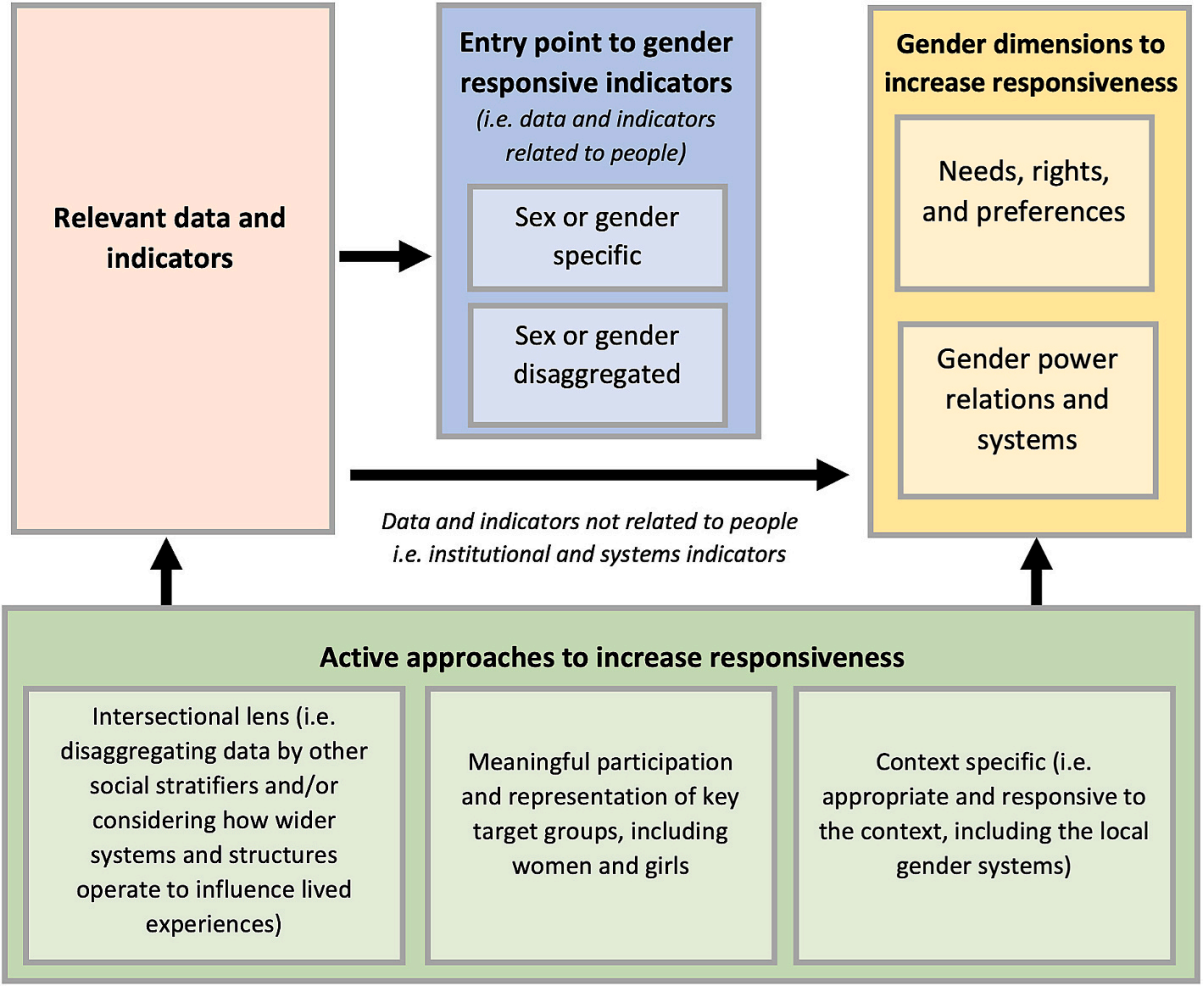
Active approaches to gender-responsive M&E adapted from Morgan et al. 2024.

Omitting sex and gender from infectious disease models downplays their impact, leading to incomplete risk assessments and ineffective, inequitable interventions. The MAGE gender-responsive M&E framework defines gender-responsive M&E as the intentional integration of the needs, rights, preferences and power relations of women and girls, men and boys, and gender minority individuals within broader social, political, economic and health systems [17]. The framework incorporates a range of gender-relevant data, including sex/gender-specific, sex/gender-disaggregated, and gender power relations and systems indicators (Fig. 1). The framework uses sex and gender labels flexibly, recognizing that indicators may reflect biological differences or socially constructed roles and norms, and applies sex/gender-specific and sex/gender-disaggregated labels to capture both; these labels serve as entry points to identify inequities while allowing adaptations across populations and contexts. While improved data collection to capture sex/gender-disaggregated health information is essential [9], the framework highlights that this can be more gender-responsive by incorporating intersectional stratification (e.g., by wealth, education, or residence) and integrating the needs, rights, and preferences of women, men, and gender minority individuals [17]. This allows for a comprehensive understanding of disparities and inequities and can help identify gender-related barriers to healthcare access, enabling more inclusive and effective interventions.

We apply the gender-responsive M&E framework developed by MAGE to provide guidance to researchers on how to structure and contextualize infectious disease modeling studies that incorporate sex/gender to better inform intervention and policy responses. In the following sections, we define five elements of the gender-responsive M&E framework and explain how they can be applied to infectious disease models. Depending on modeling constraints (e.g., data availability, computational resources), it may be infeasible to implement each suggestion. Following as many of the guidelines as possible can still make models more gender-responsive.

### 2.1 Make the population sex/gender-disaggregated or sex/gender-specific

Sex/gender-disaggregated data include two or more sex/gender groups, while sex/gender-specific data only include one group. Sex/gender groups include cisgender women, cisgender men, transgender and gender diverse individuals. Stratifying a modeled population by sex/gender aims to account for the biological and social differences in disease transmission and outcomes. The narrative review by Auderset et al. provides detailed explanations of how sex and gender can impact transition rates between disease compartments for both behavioral and biological reasons [14]. For example, different gender groups may have different recovery times due to varying immune responses, lifestyle and health behaviors, or time to diagnosis and treatment [14]. By only including one gender group, sex/gender-specific models can also account for sex/gender differences by acknowledging the unique disease dynamics of a certain sex/gender group. For example, in the United States, men who have sex with men (MSM) account for more than 80% of HIV infections among males, and transmission models may therefore focus exclusively among men and further stratify by sexual orientation [18].

### 2.2 Include an additional social stratifier

Including an additional stratifier reflects the use of an intersectional lens on sex/gender - an approach that examines sex/gender in combination with other social stratifiers, such as age, race/ethnicity, sexual orientation, education attainment, income, urbanicity, and occupation [19], [20]. This approach can uncover how these intersecting social identities shape experiences of power, vulnerability, and inequity, which can play an important role in disease transmission. In some contexts, for example, MSM may have less access to HIV prevention and treatment than men who have sex exclusively with women due to stigma and discrimination [21]. In the context of COVID-19, transmission may be higher among healthcare workers than other labor categories [22]. Including an additional social stratifier ensures that findings are not extrapolated to everyone within a sex/gender group. There might be key within-group differences, revealing compounding inequities indicating a need for targeted interventions [23]. If most onward transmissions come from men rather than women, for example, but the imbalance is only present for a narrow age group of men, it may not be an efficient use of resources to target all men with prevention efforts.

### 2.3 Maintain stratification throughout the study

Maintaining stratification from model design (population stratification) to the presentation of results (i.e., text, tables, and figures) ensures that potential confounding inequities are identified. It is common for studies to disaggregate in their analysis only to aggregate outcomes in reporting, especially when no differences are found. Even if differences among subgroups do not emerge, it is important to present these findings to clearly show there are no differences as this information can still be valuable for decision-making. Disaggregation is particularly important because we will not identify differences in outcomes if we are not looking for them.

### 2.4 Address gender dimensions

While sex/gender stratification can be an entry point into gender-responsiveness, it is not enough to make a model gender-responsive according to the MAGE criteria. The MAGE framework defines the concept of gender dimensions as the needs, rights, and preferences of gender groups, as well as gender power relations and systems (Table 1). Needs refer to the health services, programs, and care required by different gender groups to ensure their well-being. For example, MSM as well as female sex workers (FSW) have a higher risk for HIV infection, highlighting the need for prevention strategies tailored to these groups [24]. Rights encompass what individuals are entitled to in terms of healthcare and well-being, often grounded in international human rights frameworks. For instance, in the context of HIV, provision of non-discriminatory access to healthcare is especially important and reflects a human rights-based approach [25]. Preferences reflect the choices individuals make regarding their healthcare, influenced by cultural, social, or personal factors. An example of this would be ensuring that cisgender women have access to contraceptive methods of their choice at family planning facilities. Gender power relations and systems refer to the ways inequities manifest in health and health systems experiences and outcomes, such as through differential: access to resources, roles and practices, norms and beliefs, decision-making power and autonomy, and policies, laws, and institutions [19]. For example, assessing the percentage of healthcare facilities managed by women can help reveal gender disparities in leadership.

**Table 1:** MAGE gender dimension definitions and examples.

Gender dimension	Gender implications	Examples
**Needs**	• What a sex or gender group needs in terms of health services, programs, and care	• Maternal and child health services
**Rights**	• What a sex or gender group has a right to in terms of health services, programs, and care	• Universal human rights to gender equality and health (Sustainable Development Goals [SDGs])
**Preferences**	• What a sex or gender group prefers in relation to health services, programs, and care • When they are not met, they may act as barriers to access and utilization	• Private examination rooms • Women healthcare providers
**Gender power relations and systems**	• Considers the ways inequities manifest in: access to resources, roles and practices, norms and beliefs, decision-making and autonomy, and policies, laws, and institutions	• Women requiring permission to leave the house • Agency in initiating condom use

Modeling studies can address gender dimensions by explaining why disparities in health outcomes exist, for example, describing how discrimination and stigma manifest in local healthcare systems. Studies can also consider gender dimensions in the scenarios they choose to model. In a context where MSM experience stigma and discrimination, for example, self-testing may be preferred over testing at a clinic. The contextual information surrounding gender norms incorporated in modeling studies should be grounded in empirical social research on the topic. Looking across qualitative, observational, and implementation research is essential for developing a fuller understanding of the gendered context. For instance, qualitative interviews with MSM about their experiences with HIV testing offer insightful evidence on preferences, barriers, and social dynamics that could inform model structure, parameters, and scenarios [26]. Considering gender dimensions helps decision makers understand the facilitators and barriers to disease prevention, treatment, and care and can inform more impactful responses.

### 2.5 Take active approaches to gender-responsiveness

In addition to addressing gender dimensions, studies can increase gender-responsiveness by taking the active approaches defined by MAGE. MAGE defines active approaches to gender-responsiveness as including participation of key target groups and making indicators context specific. With regards to disease modeling, active approaches to gender-responsiveness can facilitate context-specific parameterization, scenario design, and validation. For example, engaging local stakeholders in choosing parameter values and in designing the model and scenarios is one way of incorporating meaningful participation from people familiar with the specific dynamics and challenges of the modeled population. Local survey data can be used to justify parameter values and situate models in context. Studies can also discuss implications for decision making by providing examples of existing policies and services and justifying specific interventions or policy recommendations. Observed local time series data for the region of interest can be used in formal calibration exercises to determine parameter values as well as validation analyses where simulated results are compared to real-world data. When time series data disaggregated by gender are unavailable, actively engaging local stakeholders and seeking observational and qualitative studies are important steps in parameterization.

The guidelines for gender-responsive infectious disease modeling are summarized in Table 2, which acts as a workflow for modelers and includes a case study example. We elaborate on additional examples in the scoping review results as well as Supplementary File 2 of included studies. The guidelines extend beyond traditional infectious disease modeling and health equity approaches by shifting from conceptual explanations of inequity to a practical measurement-focused approach that systematically integrates gender power relations and intersectional indicators within infectious disease modeling. It goes beyond simple stratification by sex or social factors to captures how intersecting identities influence infectious disease outcomes in relation to needs, rights, preferences, and the gendered systems in real-world contexts.

**Table 2:** **Guidelines for gender-responsive infectious disease models.** While stratification is an entry point, active approaches are also needed for gender-responsive infectious disease modeling according to MAGE criteria. See Supplementary File 2 for additional case study examples.

	Guideline	Justification	Examples	Case study – Bórquez et al. 2020 [27]
Stratification (entry point to gender-responsiveness)	1. Make the population sex/gender-disaggregated (two or more sex/gender groups) or sex/gender-specific (one group)	Accounts for biological and social differences in disease transmission and outcomes	Women, men, non-binary, transgender women (TW), transgender men, geographically/culturally-specific gender groups	TW and MSM
	2. Include at least one other social stratifier that is important in systems of power or transmission	Accounts for how overlapping identities shape transmission and outcomes	Age, race/ethnicity, sexual orientation, education attainment, income, urbanicity, refugee status, migrant status, injection drug use, and occupation	Stimulant use, sexual orientation, engagement in sex work
	3. Maintain stratification throughout the study	Can reveal confounding inequities and inform targeted responses	Text, tables, or figures (including appendices) showing results by different gender-age groups. Even if no differences are found, this information can inform decision making	Fig. 1 shows the ratio of the proportion of simulated new HIV infections in each group (homosexual MSM, bisexual MSM, male sex workers, TW)
Approaches for gender-responsiveness	4. Address the gender dimensions of needs, rights, preferences, and gender power relations and systems in the Background and Discussion sections	Helps decision makers understand facilitators and barriers to prevention, treatment, and care	*Needs:* What a sex or gender group *needs* in terms of health services, programs, and care (e.g., maternal healthcare)	Emphasizes the importance of policies guaranteeing the fundamental rights of transgender sex workers, such as official gender recognition, protection from violence and harassment, and the right to health and employment
			*Rights:* What a sex or gender group has a *right* to in terms of health services, programs, and care (SDGs)	
			*Preferences:* What a sex or gender group *prefers* in relation to health services, programs, and care (e.g., private examination rooms)	
			*Gender power relations & systems:* Considers how inequities manifest in access to resources, roles and practices, norms and beliefs, decision-making and autonomy, and policies, laws, and institutions (e.g., agency in initiating condom use)	
	5. Use active approaches for gender responsiveness by including participation of key target groups in model and scenario design, focusing on a specific context, and discussing intervention/policy implications	Grounds model in real-world contexts and increases decision-making relevance	*Participation of key target groups:* Engage local stakeholders and service users in model and scenario design	• Stimulant use is practiced among sex workers in Lima, Peru to cope with hunger, violence, and long hours of standing outside • Studies found lower PrEP adherence among MSM/TW who use stimulants • Model parameterized using data from local MSM/TW surveillance rounds and local epidemiological studies • Incorporating sensitive substance use screening with HIV testing, prevention, and care could facilitate access to harm reduction interventions and reduce HIV transmission
			*Context-specific:* • Explain why disparities in health outcomes exist, including cultural and geographic factors • Describe existing local policies, laws, and healthcare services • Use local survey data to parameterize the model • Use observed data for region/population of interest in calibration and/or validation	
			*Decision-making relevance:* Propose specific interventions that consider gender dimensions	

## 3 Scoping review methods

To identify studies considered gender-responsive according to the MAGE criteria, we conducted a systematic literature search following the Preferred Reporting Items for Systematic Reviews and Meta-Analysis (PRISMA) guidelines for scoping reviews [28]. A protocol was not registered for this scoping review. We searched literature for disease dynamic transmission models of COVID-19, HIV, and malaria that disaggregated the population by sex/gender and an additional social stratifier, considered gender dimensions, and took active approaches to gender-responsiveness. The following sections describe the search strategy, eligibility criteria, article selection, inclusion criteria, and data extraction and analysis.

### 3.1 Search strategy

We searched PubMed, Embase, Scopus, and Global Health in April 2024 and April 2025. Our search strategy considered three concepts: 1) dynamic transmission models, 2) the diseases of interest (COVID-19, HIV, and malaria), and 3) sex/gender. Keywords and subject headings within each concept were combined using the OR Boolean operator, and concepts were combined using the AND Boolean operator. Supplementary File 1 includes search terms for each database.

### 3.2 Eligibility criteria

We only considered published research articles, including preprints, and excluded abstracts and conference proceedings. Our search did not include any restrictions on the date the study was published, but we only reviewed studies published in English or with a published available English translation. We did not translate articles ourselves with translation software or AI. We only considered studies that used a single model and excluded reviews and comparisons of multiple models. If a study compared multiple models, we looked for the original publications of the individual models.

### 3.3 Article selection

We imported search results into the online platform Covidence [29] and followed the PRISMA guidelines for scoping reviews [28]. Title/abstract screening was conducted by seven reviewers (AH, IP, YO, JR, SS, NR, and AO). Uncertainties that arose between two reviewers during the title/abstract phase were resolved by a third reviewer or by AH. Full-text screening was conducted by four reviewers (AH, NR, IP, and YO), with each article reviewed by at least one reviewer. If the initial reviewer had any uncertainties, the article would be reviewed by a second reviewer with uncertainties discussed until a consensus was reached.

### 3.4 Inclusion criteria

To make our scoping review as systematic as possible and narrow search results to only studies applying the MAGE framework, we designed six specific inclusion criteria justified below and in Table 3. Criteria 1 and 2 pertain to dynamic transmission models of the three diseases. Criteria 3–6 are based on the MAGE framework.

**Table 3:** Inclusion criteria examples and justifications.

Criteria	Examples	Justification
1. Dynamic transmission model	• Compartmental, agent-based, Markov, System Dynamics, Discrete Event Simulation	• Many HIV models are agent-based models (ABMs) • Many COVID-19 and malaria models are compartmental • A way to narrow the search (excluded statistical time series models)
2. Summary measure of COVID-19, HIV, or malaria	• Cases • Deaths • Hospitalizations • Reproduction number	• Representation of respiratory, vector-borne, and sexually transmitted diseases • Common in forecasting and scenario modeling • Good examples in HIV literature with transferable aspects to COVID-19 and malaria • Narrow search (e.g., excluded mental health outcomes)
3. Sex/gender disaggregated population	• Different compartments and parameters for cisgender women, cisgender men, transgender or gender diverse individuals • Separate model runs for different gender groups • Agents with sex/gender attribute (ABMs)	• Make COVID-19, HIV, and malaria studies more comparable (e.g., unlikely to find gender-specific COVID-19 model) • Expose differences between sex/gender groups • Narrow search (excluded sex/gender-specific model populations)
4. Additional social stratifier in at least one sex/gender group	• Age- and gender-structured compartmental model • ABM with agent attributes for gender and race/ethnicity • Other stratifiers considered: sexual orientation, education attainment, income, urbanicity, refugee status, injection drug use, occupation (e.g., healthcare workers, sex workers)	• Identities that are important in systems of power and/or transmission • Intersectional lens (otherwise treating everyone the same) • Can reveal compounding inequities • Important for resource allocation and targeting interventions to certain groups
5. Stratification maintained throughout study	• Simulated infections/deaths for gender-age or gender-race groups in text, tables, or figures	• Not enough to stratify population in methods/model design • Need to see results for subgroups to inform decisions, even if no differences are found
6. Considers gender dimensions of needs, rights, preferences, and gender power relations and systems	• Thought out in detail in Introduction or Discussion sections • Justification for model or scenario design • Propose interventions/policies that take needs, rights, and preferences into account	• Attempt at explaining why differences exist • Considers context (e.g., presence or absence of health services, programs, policies, or laws) • Important for designing interventions/policies that meet needs, rights, and preferences

#### Criteria 1: Dynamic transmission model

Infectious disease models fall into two categories: disease dynamic and statistical. Disease dynamic models, also referred to as epidemiological or mechanistic models, attempt to explain the mechanisms of disease transmission within model equations. [30]. The classic compartmental susceptible, infected, recovered (SIR) model, for example, uses a system of ordinary differential equations (ODEs) in which infection is governed by the transmission rate [31]. In an agent-based model (ABM), transmission is a function of the number of contacts per agent and the probability of infection per contact. Statistical models (e.g., ARIMA, machine learning) do not have rules or equations governing transmission. Instead, they extrapolate from past data, usually using regression models, to predict future trends in outcomes [30]. To narrow our search, we chose to focus on disease dynamic models, including compartmental, agent-based, system dynamics, Markov, and discrete even simulation models. We also only considered transmission models and excluded studies that did not simulate new infections (i.e., a fraction of the population needed to begin the simulation as susceptible). We therefore excluded HIV studies where the initial population was entirely infected, as well as studies that modeled outcomes besides infections (e.g., length of stay or antiretroviral therapy [ART] adherence) if they did not include a mechanism for transmission as well.

#### Criteria 2: COVID-19, HIV, and malaria

We focused on three diseases (COVID-19, HIV, and malaria) because they represent distinct transmission routes (respiratory, sexually-transmitted, and vector-borne) that collectively account for a substantial portion of global infectious disease burden and receive significant modeling and policy attention. Each disease has existing literature describing how sex and gender can influence outcome distributions. We hoped this diversity would enable a robust assessment of how gender can be incorporated across different transmission pathways and health systems contexts.

Historically, HIV models have considered gender with regards to disease dynamics much more so than COVID-19 or malaria. We hypothesized the HIV literature would provide useful examples with transferable aspects for other diseases. Model outcomes were restricted to summary measures (e.g., infections, deaths, hospitalizations, reproduction number) because these are commonly simulated among the three diseases, and this made the studies included in our review more comparable. We excluded studies that simulated other outcomes (e.g., mental health or economic outcomes) unless they also presented summary measures of infectious disease.

#### Criteria 3: Sex/gender-disaggregated population

Included models needed to stratify the population by two or more sex/gender groups, such as cisgender women, cisgender men, transgender or gender diverse individuals. We excluded sex/gender-specific populations to make models of the different diseases more comparable. For instance, there are many sex/gender-specific HIV models that could be considered gender-responsive according to our guidelines, but there are very few COVID-19 studies where the modeled population consists of only one gender group.

#### Criteria 4: Additional social stratifier

Following the MAGE framework, we required models to disaggregate the population by an additional social stratifier as well as sex/gender as a way to take an intersectional lens. We considered the following social stratifiers: age, race/ethnicity, sexual orientation, education attainment, income, urbanicity, refugee status, migrant status, injection drug use, and occupation. While not all-encompassing, these identifiers are important in systems of power and transmission.

#### Criteria 5: Stratification maintained

Studies needed to maintain stratification by sex/gender and the additional social stratifier throughout the study by presenting simulation results for the different groups in text, tables, or figures, including appendices.

#### Criteria 6: Gender dimensions and active approaches

While disaggregating the population is an important step in incorporating a gender lens, it is not enough to make a model gender-responsive according to the MAGE framework. Our sixth criteria looked at how studies addressed the gender dimensions of needs, rights, preferences, and gender power relations and systems (Table 1) and how they used the active approaches for gender-responsiveness (Fig. 1). Included studies needed to explain why disparities in health outcomes existed and put their research in context by describing the local healthcare and policy landscape, engaging local stakeholders in model or scenario design, using local survey data in parameterization, or comparing simulation results to observed data for their region of interest. We also required studies to discuss implications for decision making by justifying specific interventions or policy recommendations given their findings.

We conducted screening iteratively, first identifying dynamic transmission models of the three diseases in the title/abstract phase. During full-text screening, we narrowed these down to studies that stratified the population by sex/gender. Then we identified studies that disaggregated by an additional social stratifier and maintained stratification throughout the results. Finally, we filtered these studies to those that addressed gender dimensions and used active approaches for gender-responsiveness.

### 3.5 Data charting

Data were extracted in an Excel spreadsheet (Supplementary File 2) using a charting form developed by the authors. For each study, we extracted the title, author, journal, year published, model type, study population location and stratification, scenarios modeled, and consideration of gender dimensions and active approaches. We used ChatGPT (version 4) to conduct the initial round of data extraction by uploading an article PDF and prompting ChatGPT to create a spreadsheet based on provided column descriptions. After producing the first row of the spreadsheet, ChatGPT was prompted to add additional rows by uploading one study at a time. This process is described in more detail in Supplementary File 1. ChatGPT did well extracting basic article information (title, authors, year published, disease modeled) but lacked detail with columns requiring more nuance. For example, ChatGPT sometimes hallucinated reasons authors cited for why disparities existed in the context of their study. Every article was read in-full with data extracted manually by at least one reviewer. If the initial reviewer had any uncertainties, a second reviewer would also read the article, with uncertainties discussed until consensus was reached.

### 3.6 Data analysis

We categorized the analysis into three key areas: 1) summary statistics of diseases modeled, model types, projected outcomes, additional social stratifiers, and scenarios modeled; 2) a narrative synthesis of the incorporation of gender dimensions; and 3) a narrative synthesis of how studies adopted active approaches to gender-responsiveness. We did not conduct quality appraisal of included studies.

## 4 Scoping review results

### 4.1 Search results

**Fig. 2:**
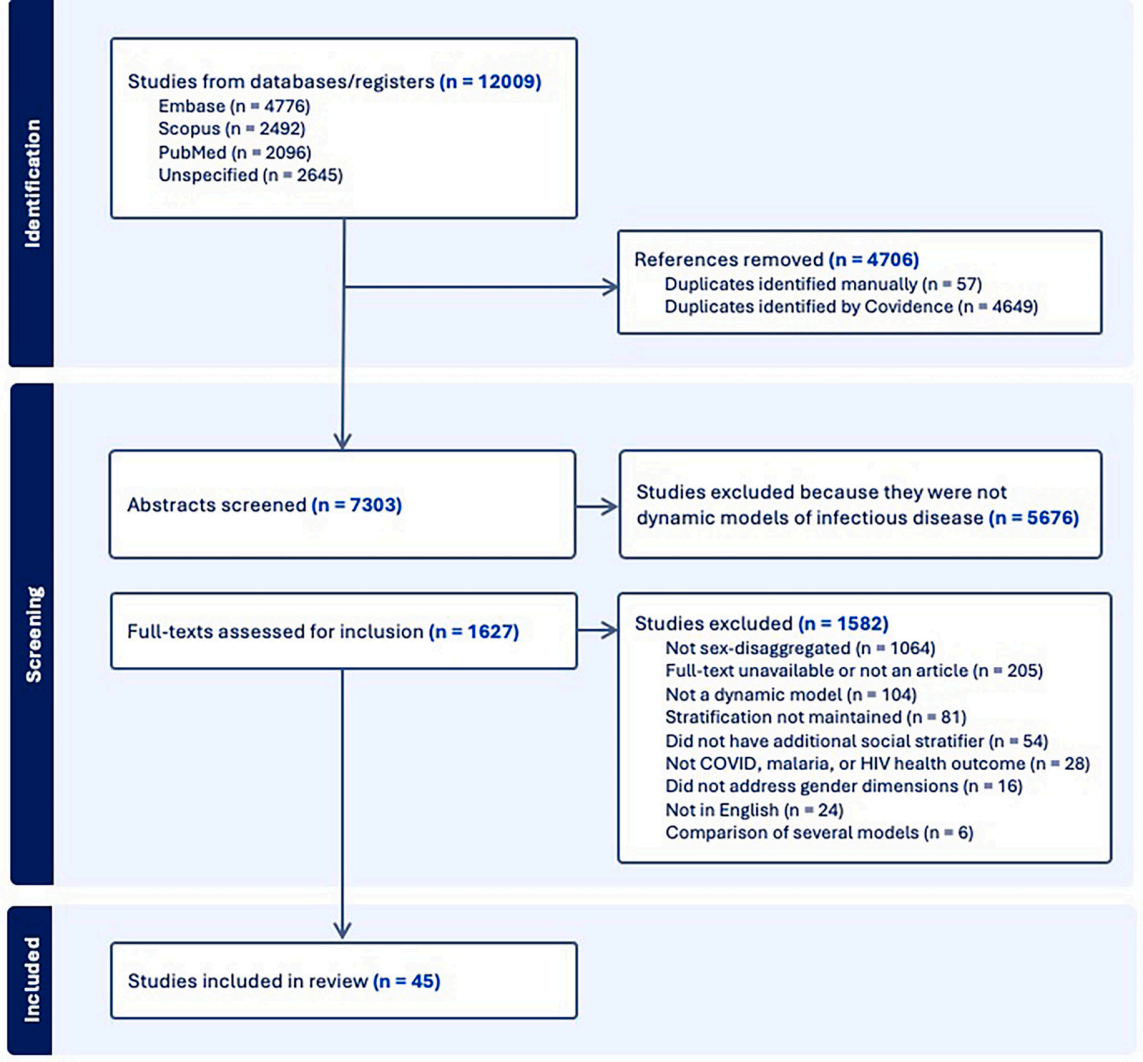
PRISMA flow chart.

**Fig. 3:**
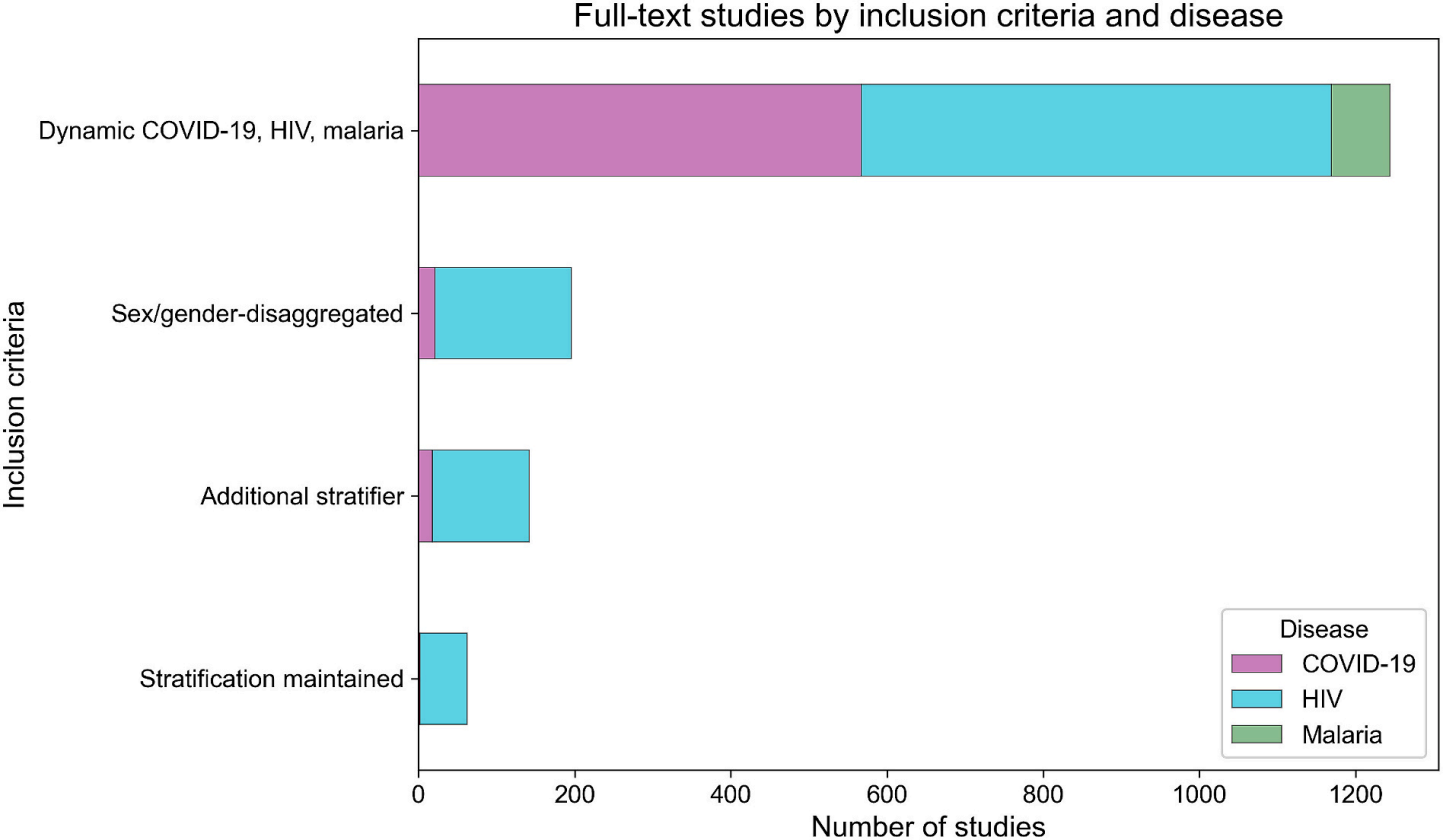
Full-text studies by inclusion criteria and disease. Full-text screening was conducted iteratively, and we first identified dynamic transmission models of COVID-19, HIV, or malaria (top bar, *N* = 1243). We then filtered for sex/gender-disaggregated studies (second bar from top, *N* = 195), then for studies that included an additional social stratifier (third bar from top, *N* = 141), and then for studies that maintained stratification throughout the study (bottom bar, *N* = 61). In each iteration, most studies were HIV models (blue). We did not identify any sex/gender-disaggregated malaria models (green). We identified two COVID-19 models (purple) and 59 HIV models that maintained stratification. We then filtered these for studies that addressed gender dimensions and included active approaches (not shown). (For interpretation of the references to colour in this figure legend, the reader is referred to the web version of this article.)

Database searches returned a total of 12009 abstracts, of which 4706 were duplicates and removed. Title and abstract screening included 7303 studies, among which 5676 were irrelevant. Among the 1627 identified for full-text screening, 132 were excluded because they were not dynamic transmission models of one of the three diseases, 1064 were excluded because they were not sex/gender-disaggregated, 54 did not include an additional social stratifier, 81 did not maintain stratification, and 16 did not address gender dimensions. Two hundred twenty-nine studies were excluded because they were not articles or full-texts, or English translations were unavailable. Forty-five studies were included in the final review (Supplementary File 2 of included studies): 43 simulated HIV, and two simulated COVID-19. We identified 108 malaria studies for full-text screening; however, 22 were conference abstracts or did not have full-texts available, and nine were excluded because they were not a dynamic model simulating an infectious disease summary statistic. The remaining 77 were excluded because they were not sex/gender-disaggregated. Fig. 2 presents the PRISMA flow chart, and Fig. 3 shows the number of full-text studies by inclusion criteria and disease.

### 4.2 Summary statistics

Among the 45 included studies, 43 modeled HIV transmission and two modeled SARS-CoV-2 transmission (Table 4); we did not identify any malaria models that met all inclusion criteria. Thirty-three studies used compartmental models, ten used agent-based models, one used a Monte-Carlo simulation, and one used a discrete event simulation. In addition to projecting infections, nine models simulated deaths, four estimated the reproduction number, and eight simulated changes in coverage of prevention, testing, or treatment. Ten studies included a cost-utility or cost-effectiveness outcome. Sexual orientation (*N* = 23) and occupation (*N* = 23) were the most frequent additional social stratifiers. Most studies that stratified by occupation simulated HIV transmission and included a separate compartment for commercial sex workers. One COVID-19 study stratified the population by labor sector. Other additional social stratifiers included age (*N* = 17), drug use (*N* = 9), urbanicity (N = 2), and race/ethnicity (N = 1). Twenty-nine HIV studies modeled prevention scenarios (i.e., condom use, PrEP, vaccination, or voluntary medical male circumcision (VMMC), 16 modeled scenarios varying ART coverage, and eleven modeled scenarios varying testing coverage. Eleven studies varied contact mixing patterns in scenario analysis, and six studies modeled different scenarios of education, awareness, and harm reduction (e.g., needle exchange). Twenty-two studies modeled populations in sub-Saharan Africa, one in northern Africa and the Middle East, eleven in Asian countries, one in Oceania, one in South America, two in the United Kingdom, two in Europe, five in the United States, and one in Canada. Table 4 presents summary statistics of included studies.

**Table 4:** Summary statistics of included studies (*N* = 46).

Model type	**N**	**%**
Compartmental	33	73%
Agent-based	10	22%
Monte-Carlo	1	2%
Discrete Event Simulation	1	2%

**Disease**	**N**	**%**

COVID-19	2	4%
HIV	43	96%

**Projected outcomes**	**N**	**%**

Infections	45	100%
Deaths	9	20%
Reproduction number	4	9%
Costs or cost-utlity/effectiveness	10	22%
Coverage of prevention, testing, or treatment	8	18%

**Additional social stratifier**	**N**	**%**

Age	17	38%
Sexual orientation	23	51%
Occupation	23	51%
Drug use	9	20%
Race/ethnicity	1	2%
Urbanicity	2	4%

**Scenarios Modeled**	**N**	**%**

PrEP	8	18%
Condom use	12	26%
Voluntary medical male circumcision (VMMC)	5	11%
Testing	11	24%
ART	16	36%
Vaccination	4	9%
Contact mixing patterns	11	24%
NPIs (COVID-19)	2	4%
Other (e.g., education, awareness, reduced or safer drug use)	6	13%

### 4.3 Stratification

All the compartmental models used population stratification to operationalize differences in behavior between sex/gender subgroups. In the model by Johnson et al., for example, the HIV transmission probability per unprotected sex act in short-term relationships from male-to-male was 0.020 compared to 0.008 for female-to-male [26]. Several HIV studies used different testing and ART coverage and retention rates by subgroup based on gender, sexual orientation, and occupation (sex work). The COVID-19 model by Doerre et al. used a sex-age contact matrix, where higher contact rates among women led to higher transmission rates among women compared to men in simulations [32]. ABMs used real-world demographic data to generate synthetic networked populations. For example, Manout et al. used Canadian Census and various data sources to connect agents with gender and age attributes in home, school, and workplace networks; they applied higher probabilities of developing serious infection to older age groups [22]. Hamilton et al. used survey data to construct heterosexual and MSM sexual networks with agents with gender, age, and race attributes [33]. The synthetic population aimed to preserve key features of the surveyed population, including the number of partnerships between different age, gender, race, and sexual orientation groups.

### 4.4 Gender dimensions

To be gender-responsive according to our criteria, studies needed to address one or more of the gender dimensions defined in the MAGE framework. The following sections comprise a narrative review of how included studies incorporated needs, rights, preferences, and gender power relations and systems.

#### 4.4.1 Needs and rights

Often needs were discussed by explaining factors that led to gaps in care and unmet treatment needs. For example, Stone et al. explained that the historical focus on heterosexual HIV transmission in African settings has led to unmet prevention and treatment needs for key populations, including MSM and FSW [34]. Chemaitelly et al. discussed how stigma, discrimination, and punitive laws dissuade these groups from seeking testing and treatment in the Middle East and Northern Africa [21]. Bórquez et al. emphasized the importance of policies guaranteeing the fundamental rights of transgender sex workers, such as official gender recognition, protection from violence and harassment, and the right to health and employment [27].

#### 4.4.2 Preferences

Several studies described the preferences of a sex/gender group with regards to interventions and health services. Kripke et al. explained that a lack of male service providers and discrete services can act as a barrier to VMMC [35]. In models exploring HIV testing and treatment strategies in West Africa, Silhol et al. and Johnson et al. explained that self-testing could avoid stigma and discrimination experienced by marginalized groups [36], [37]. With regards to treatment, Johnson et al. noted that local surveys suggested preferences for injectable versus oral PrEP [26].

#### 4.4.3 Gender power relations and systems

Studies discussed gender power relations and systems in the context of sexual partnership formation, marriage practices, and gender-biased job markets. Several HIV studies set in African countries, for example, cited age differences between men and women in sexual partnerships, where men tend to be older, as a reason for higher HIV incidence in young women compared to young men [38], [39], [40], [41], [42], [43]. In these studies, age was included as an additional social stratifier with different parameter values for different age groups. Some studies went a step further and discussed women's economic dependence on men in these settings [44] and their lack of agency in negotiating safe sex practices [38], [45]. One COVID-19 study, and nine HIV studies, listed varying occupational exposure as a reason for gender disparities. The COVID-19 study discussed how jobs in healthcare and education are more often filled by women, migrants, and low-wage workers in Montreal, Canada, and these labor sectors experienced higher disease burden in the early phase of the pandemic [22]. HIV studies that focused on occupation mentioned risk behaviors, including drug use and unsafe sex, by FSW and migrant workers [27], [44], [46].

### 4.5 Active approaches to gender-responsiveness

Active approaches to gender-responsiveness include being context specific and incorporating participation from key target groups. Studies in our review used active approaches by explaining why disparities existed, incorporating participation from local stakeholders, describing local healthcare services, policies, and laws, and detailing decision-making implications.

#### 4.5.1 Explanation of disparities

One way studies took an active approach to gender-responsiveness was by being very context specific in their explanation of the factors leading to health disparities. For example, Bórquez et al. explained that stimulant use is a practice among street sex workers in Lima, Peru to cope with hunger, violence, and long hours of standing outside [27]. Deering et al. explained that truckers and migrant workers in Karnataka, India who experience loneliness and separation from families may be more likely to seek out commercial sex [46].

Researchers included participation from relevant stakeholders by engaging them in model and scenario design. For example, Selinger et al. conducted individual interviews with government, academic, and community stakeholders to parameterize their model and ensure their analysis reflected the realities of the South African HIV epidemic [47]. They also held a vaccine access planning summit and modeling workshop where they shared preliminary findings and elicited feedback. As an indirect way of including participation from key target groups, several studies parameterized their models using local survey and interview data [21], [26], [34], [48], [49], [50].

#### 4.5.2 Description of current intervention and policy landscape

Another way studies were context specific was by describing existing interventions, policies, and gender power relations and systems specific to their region of interest. Omondi et al. noted innovative approaches in Kenya's HIV response, including self-testing kits, door-to-door testing campaigns, and targeted community-based testing [38]. Mishra et al. explained that a lack of rapport between healthcare staff and MSM acts as a barrier to HIV prevention in Mumbai, India [48]. Chemaitally et al. explained that HIV prevention and treatment in the Middle East and North Africa is largely led by under-resourced non-profit organizations [21].

#### 4.5.3 Decision-making relevance

To be included in our review, studies needed to contextualize implications for decision making. Researchers did this by suggesting interventions or policies that targeted marginalized groups [21], [22], [33], [34], [35], [36], [37], [38], [39], [41], [42], [45], [46], [48], [49], [50], [51], [52], [53], [54], [55], [56], [57], reduced stigma and discrimination [21], [36], [37], [54], [56], addressed social determinants and structural barriers [26], [32], [51], [58], [59], or prioritized one type of intervention over another [37], [40], [44], [45], [49], [60], [61], [62]. For example, rather than blanket COVID-19 restrictions for all workplaces, Manout et al. suggested that a more tailored response in addressing needs in healthcare and education sectors might better balance the health-economic tradeoffs faced during respiratory epidemics [22]. Similarly, Hamilton et al. explained that racial disparities would persist with universal expansion of HIV testing and treatment programs and advocated for a more targeted approach [33]. Studies also noted the need for more disaggregated data to facilitate scenario modeling and analyze the impact of intervention and policy changes on population subgroups. Bórquez et al. provided an excellent example of situating a mathematical model in context with a thorough description of existing interventions and a box of policy implications [27].

## 5 Discussion

Incorporating sex and gender into infectious disease models can improve their predictive accuracy, help understand heterogeneity in disease burden and policy impact, contextualize decision making, and address inequalities and implementation challenges [16], [63]. There is increasing recognition that sex and gender play important roles in infectious disease transmission, but there remains a lack of guidance for modelers on how to incorporate a gender-responsive lens. To address this gap, we developed specific guidelines for gender-responsive infectious disease models informed by an established gender analysis framework and conducted a scoping review to illustrate their application.

The guidelines for gender-responsive models serve as a structured approach to enhance the interpretability of infectious disease models by embedding context and decision-making relevance at every stage. Our guidelines help bridge the gap between epidemiological models and public health decision-making and can be applied to other forms of disparities. For example, Tulchinsky et al. use an agent-based model with a population stratified by race and socio-economic status to show how school and workplace closures can lead to disparities during respiratory epidemics like COVID-19 [64].

Due to data availability and limits on computational power, modelers must often be selective in how they stratify a population. When there is an urgent need to forecast burden quickly (e.g., early pandemic), stratifying by gender (or other social identifiers) may be infeasible within time constraints. In contrast, including social stratifiers may be very important in scenario modeling that aims to test the impact of different interventions or inform limited resource allocation. Open-source agent-based models and synthetic population generators can facilitate population stratification. For example, modeling tools like Starsim [65], EMOD [66], and EpiModel [67] allow population disaggregation with subgroup-specific disease parameters. Such models can be run on synthetic populations built from Census or survey data (see Manout et al. [22] and Hamilton et al. [33] for examples from this review) to more realistically approximate outcomes by demographic and geographic groups. Software packages that generate Census-based synthetic populations, such as GeoPops [68] and UrbanPop [69], have been developed for the US [68], [69], [70], [71], [72], Canada [22], France [73], and China [74], [75], although this list is not exhaustive.

Findings from our scoping review highlight best practice examples of incorporating a gender-responsive lens, mainly from HIV literature, that can be useful for other diseases. Among the 45 identified studies, most were compartmental and agent-based models of HIV and included sexual orientation or occupation as an additional social stratifier. Two COVID-19 models were identified, and no malaria models met our inclusion criteria. HIV has long been recognized as a disease with strong gender dimensions, with well-documented disparities in infection risk and treatment access and uptake. As evidenced by this review, relevant HIV data disaggregated by sex, sexual orientation, and occupation exist in low- and middle-income countries, which often do not have disaggregated data for other diseases. In contrast, COVID-19 models emerged rapidly in response to the pandemic, with an urgent focus on forecasting overall burden and a prioritization of broad public health measures (e.g., lockdowns, vaccinations) rather than targeted interventions addressing gender. Malaria modeling has traditionally focused on vector dynamics, climate factors, and drug resistance, although there is growing documentation of how gendered behavioral factors can drive transmission. For example, among the nine malaria studies that were excluded from full-text screening in this review because they were not disease dynamic models simulating an infectious disease summary measure, four identified differences in movement patterns and malaria outcomes by gender and age using statistical and network approaches [76], [77], [78], [79]. Three of the excluded studies examined the impact of prevention efforts (bed nets, vaccination, and chemoprevention) during pregnancy on neonatal mortality [80], [81], [82].

Findings also highlight an underrepresentation of other social stratifiers that compound gender inequities, such as disability, migration status, caste, and socio-economic status, in infectious disease modeling studies. Similarly, a previous scoping review found that age, poverty, and sex were the most commonly incorporated stratifiers in infectious disease compartmental models, while cultural determinants were often excluded, likely reflecting limited data availability compared with routinely collected variables, such as age and sex [83]. Methods such as sensitivity analyses, proxy variables, and Bayesian approaches can help when disaggregated data are unknown or uncertain [84]. Sensitivity analyses can help quantify the impact of assumed and uncertain parameter values (See [38], [42], [54], [56] for examples from this review). Proxy variables can help a model maintain stratification when disaggregated data are unavailable; for example, if contact rates by sex/gender are unknown, cellphone mobility data by sex/gender can inform assumptions about disaggregated contact rates. Bayesian approaches can also help when data are unavailable for different population groups by assigning a distribution around a parameter rather than a fixed number to reflect uncertainty;or example, occupation-specific contact rates could be modeled as a distribution around a mean rather than having a different contact rate for each occupation. In this review, Silhol et al. [36] used a Bayesian framework to parameterize and fit their model to country-specific outcomes by sex, risk, and age group.

### 5.1 Limitations and future research

We aimed to conduct a specific yet comprehensive scoping review; however, our review has some limitations. First, our search strategy was restricted to English-language publications, and we likely missed relevant malaria models published in French from endemic Francophone regions in West Africa. Second, our inclusion criteria were intentionally restrictive to narrow search results and follow the MAGE framework. We specifically looked for studies that offered examples of how the MAGE framework could be applied to infectious disease modeling. Just because a study did not meet our criteria does not mean it is not gender-responsive, and there may be many well-conducted, gender-informed infectious disease models that would not have qualified for this review. We excluded data-driven statistical models, studies where the population was sex/gender-specific, and studies that exclusively modeled non-disease outcomes (e.g., mental health or economic outcomes). Such studies could certainly be ‘gender-responsive’ and provide valuable insights for decision making with regards to gender. Reviewing other study types could help inform how gender can be incorporated into dynamic disease models outside sexually transmitted infections, which are already well-represented in the dynamic modeling literature.

Additionally, our review is likely biased toward studies that looked for and identified gender disparities. Studies that did not find differences may have aggregated results, making them ineligible for inclusion. We did not conduct quality appraisal of included studies and cannot comment on their methodological rigor or simulation accuracy. Included studies offer best practice examples of how to apply the MAGE framework in terms of stratification and increasing gender-responsiveness by contextualizing models with gender dimensions and active approaches. Studies that aim to apply the gender-responsive guidelines in this review should also aim to be methodologically robust with regards to model design, parameterization, validation, and sensitivity analysis. To test if sex/gender-disaggregation improves accuracy of simulated results, future research should compare results with and without disaggregation to real-world data.

The over-representation of HIV literature in our review may limit the generalizability of our framework to other diseases where disaggregated data are unknown or uncertain. However, as disaggregated data become more available for other diseases, the gender-responsive guidelines and HIV examples from this review can act as a roadmap for future research. Studies in our review primarily disaggregated populations by the binary gender categories of cisgender men and cisgender women, reflecting a major lack of dynamic disease models considering transgender and gender diverse populations. These populations often face unique health risks and structural barriers that impact disease burden [85], [86]. Improved data availability and quality are needed to make models truly inclusive of under-served populations, not just those based on gender [85]. We encourage modelers to proactively seek local surveys or qualitative studies to parameterize and validate models that include under-served populations that may not be represented in traditional data streams (e.g., the Census or government health outcome reporting). Modelers need to balance using observed quantitative data to justify parameters and assumptions while not excluding entire population groups because data are unavailable. In such instances, using data from qualitative, observational, and implementation research with methods such as sensitivity analyses, proxy variables, and Bayesian approaches can inform more inclusive models.

## 6 Conclusion

With structured guidelines and example studies, we provide a roadmap for making models gender-responsive and actionable for decision makers. While our guidelines focus on gender disparities, the same approach can be extended to address other forms of health inequity, including those based on race/ethnicity, income, occupation, or geography. To accelerate progress toward equity in infectious disease modeling, modelers, funders, and public health institutions must prioritize the collection and use of disaggregated data and ensure that social determinants of health are integrated into modeling frameworks. As we prepare for future and routine infectious disease challenges, models that account for the complex interplay of gender, power relations, and social contexts will be better equipped to guide effective, efficient, and equitable responses.

The following are the supplementary data related to this article.

Supplementary material 1 **S1 File.** Search terms by database and methods for using ChatGPT in data extraction

Supplementary material 2 **S2 File.** Spreadsheet of included studies with extracted data.

## Declaration of generative AI and AI-assisted technologies in the manuscript preparation process

ChatGPT 4.0 was used for initial data extraction (See Supplementary File 1 for a detailed description). ChatGPT did well extracting basic article information (title, year, publication, authors) but did not do well answering more nuanced prompts. For example, sometimes ChatGPT hallucinated reasons the article discussed for why sex/gender disparities existed in the context of the study, confusing one article with another. Every article was read in-full and reviewed manually by at least one, often two, authors to ensure the accuracy of extracted data in the manuscript text and Supplementary File 2 of included studies.

## CRediT authorship contribution statement

**Alisa Hamilton:** Writing – review & editing, Writing – original draft, Visualization, Project administration, Methodology, Formal analysis, Data curation, Conceptualization. **Indira Prihartono:** Writing – review & editing, Writing – original draft, Methodology, Formal analysis, Conceptualization. **Yamato Okura:** Writing – review & editing, Formal analysis. **Naomi Rankin:** Writing – review & editing, Formal analysis. **Samee Saiyed:** Writing – review & editing, Formal analysis. **Jeremy Ratcliff:** Writing – review & editing, Formal analysis. **Ayo Oladimeji:** Writing – review & editing, Formal analysis. **Tak Igusa:** Writing – review & editing, Supervision. **Lauren Gardner:** Writing – review & editing, Supervision, Funding acquisition, Conceptualization. **Rosemary Morgan:** Writing – review & editing, Supervision, Methodology, Funding acquisition, Conceptualization.

## Ethics statement

Ethnical approval was not required for this scoping review.

## Declaration of competing interest

Rosemary Morgan and Indira Prihartono report financial support was provided by Gates Foundation. Alisa Hamilton and Lauren Gardner report financial support was provided by Centers for Disease Control and Prevention. If there are other authors, they declare that they have no known competing financial interests or personal relationships that could have appeared to influence the work reported in this paper.
