# Supplementary material for: Gender-responsive infectious disease models: Guidelines and systematic scoping review
Source: Dialogues Health. 2026 Jul 22;9:100329. doi: 10.1016/j.dialog.2026.100329 (PMC13471233; doi:10.1016/j.dialog.2026.100329)
Supplement: Supplementary file 1 [file mmc1.docx]

**Supplement**

# Title

Gender-responsive infectious disease models: Guidelines and systematic scoping review

# Authors

Alisa Hamilton, MS, MA^1^

Indira Prihartono, MD, MHA, MPH^2^

Yamato Okura^1^

Naomi Rankin, MS^1^

Samee Saiyed, MS^1^

Jeremy Ratcliff, PhD^1,3^

Ayo Oladimeji, MS^1^

Tak Igusa, PhD^1^

Lauren Gardner, PhD^1^

Rosemary Morgan, PhD^2^

^1^Whiting School of Engineering, Johns Hopkins University

^2^Bloomberg School of Public Health, Johns Hopkins University

^3^Johns Hopkins University Applied Physics Laboratory

# Corresponding Author

Alisa Hamilton, MS, MA

Whiting School of Engineering

Johns Hopkins University

E: ahamil33@jh.edu

T: +1 (207) 944-3211

# Full search strategy by database

## PubMed

| **Concept** | **Search terms** |
| --- | --- |
| COVID-19, HIV, or malaria | ("COVID19"[Title/Abstract] OR "COVID 19"[Title/Abstract] OR "Coronavirus"[Title/Abstract] OR "SARS-CoV-2"[Title/Abstract] OR "Malaria*"[Title/Abstract] OR "Plasmodium falciparum"[Title/Abstract] OR "P. falciparum"[Title/Abstract] OR "Plasmodium vivax"[Title/Abstract] OR "P. vivax"[Title/Abstract] OR "Plasmodium ovale"[Title/Abstract] OR "P. ovale"[Title/Abstract] OR "Plasmodium malariae"[Title/Abstract] OR "P. malariae"[Title/Abstract] OR "Plasmodium knowlesi"[Title/Abstract] OR "P. knowlesi"[Title/Abstract] OR "Human Immunodeficiency Virus"[Title/Abstract] OR "HIV "[Title/Abstract] OR "HIV/AIDS"[Title/Abstract] OR "COVID-19"[MeSH] OR "SARS-CoV-2"[MeSH] OR "Malaria"[MeSH] OR "HIV"[MeSH]) AND |
| Sex/gender | ("women*"[Title] OR "girl*"[Title] OR "female*"[Title] OR "men"[Title] OR "men s"[Title] OR "boy s"[Title] OR "male*"[Title] OR "gender*"[Title/Abstract] OR "sexbased"[Title/Abstract] OR "sex based"[Title/Abstract] OR "two spirit"[Title/Abstract] OR "nonbinary"[Title/Abstract] OR "non binary"[Title/Abstract] OR "genderqueer"[Title/Abstract] OR "genderfluid"[Title/Abstract] OR "transgender*"[Title/Abstract] OR "age structure"[Title/Abstract] OR "age specific"[Title/Abstract] OR "age distribution"[Title/Abstract] OR "age stratif*"[Title/Abstract] OR "stratified by age"[Title/Abstract] OR "stratified on age"[Title/Abstract] OR "age group*"[Title/Abstract] OR "subpopulations by age"[Title/Abstract] OR "decision making"[Title/Abstract] OR "empowerment"[Title/Abstract] OR "autonomy"[Title/Abstract] OR "self efficacy"[Title/Abstract] OR "transformative"[Title/Abstract] OR "intersectionality"[Title/Abstract] OR "Sex Characteristics"[MeSH] OR "Gender Equity"[MeSH] OR "Gender Role"[MeSH] OR "Gender Identity"[MeSH] OR "Sexual and Gender Minorities"[MeSH] OR "Intersectional Framework"[MeSH] OR "Empowerment"[MeSH] OR "Personal Autonomy"[MeSH] OR "Self Efficacy"[MeSH] OR "Decision Making"[Mesh:NoExp]) AND |
| Dynamic transmission model | ("ODE"[Title/Abstract] OR "differential equation*"[Title/Abstract] OR "ABM"[Title/Abstract] OR "agent based model*"[Title/Abstract] OR "mathematical model*"[Title/Abstract] OR "compartmental model*"[Title/Abstract] OR "SIR model*"[Title/Abstract] OR "susceptible infected recovered"[Title/Abstract] OR "SEIR model*"[Title/Abstract] OR "susceptible exposed infected recovered"[Title/Abstract] OR "susceptible infected susceptible"[Title/Abstract] OR "dynamic model*"[Title/Abstract] OR "dynamical model*"[Title/Abstract] OR "Markov model*"[Title/Abstract] OR "Markov chain"[Title/Abstract] OR "network analysis"[Title/Abstract] OR "network model*"[Title/Abstract] OR "system dynamic*"[Title/Abstract] OR "epidemic model*"[Title/Abstract] OR "pandemic model*"[Title/Abstract] OR "epidemiological model*"[Title/Abstract] OR "disease dynamic*"[Title/Abstract] OR "disease transmission*"[Title/Abstract] OR "Social Network Analysis"[MeSH] OR "Stochastic Processes"[MeSH] OR "Epidemiological Models"[MeSH] OR "Systems Analysis"[Mesh:NoExp]) |

## Embase

| **Concept** | **Search terms** |
| --- | --- |
| COVID-19, HIV, or malaria | ("COVID19":ab,ti OR "COVID 19":ab,ti OR "Coronavirus":ab,ti OR "SARS-CoV-2":ab,ti OR "Malaria*":ab,ti OR "Plasmodium falciparum":ab,ti OR "P. falciparum":ab,ti OR "Plasmodium vivax":ab,ti OR "P. vivax":ab,ti OR "Plasmodium ovale":ab,ti OR "P. ovale":ab,ti OR "Plasmodium malariae":ab,ti OR "P. malariae":ab,ti OR "Plasmodium knowlesi":ab,ti OR "P. knowlesi":ab,ti OR "Human Immunodeficiency Virus":ab,ti OR "HIV ":ab,ti OR "HIV/AIDS":ab,ti OR 'coronavirus disease 2019'/exp OR 'Severe acute respiratory syndrome coronavirus 2'/exp OR 'malaria'/exp OR 'Human immunodeficiency virus'/exp) AND |
| Sex/gender | ("women*":ti OR "girl*":ti OR "female*":ti OR "men":ti OR "men s":ti OR "boy s":ti OR "male*":ti OR "gender*":ab,ti OR "sexbased":ab,ti OR "sex based":ab,ti OR "two spirit":ab,ti OR "nonbinary":ab,ti OR "non binary":ab,ti OR "genderqueer":ab,ti OR "genderfluid":ab,ti OR "transgender*":ab,ti OR "age structure":ab,ti OR "age specific":ab,ti OR "age distribution":ab,ti OR "age stratif*":ab,ti OR "stratified by age":ab,ti OR "stratified on age":ab,ti OR "age group*":ab,ti OR "subpopulations by age":ab,ti OR "decision making":ab,ti OR "empowerment":ab,ti OR "autonomy":ab,ti OR "self efficacy":ab,ti OR "transformative":ab,ti OR "intersectionality":ab,ti OR 'sexual characteristics'/exp OR 'female by marital status'/exp OR 'female by occupation'/exp OR 'female by sexual orientation'/exp OR 'male by marital status'/exp OR 'male by sexual orientation'/exp OR 'sexual and gender minority'/exp OR 'personal autonomy'/exp OR 'intersectionality'/exp OR 'empowerment'/exp OR 'family decision making'/exp OR 'gender equity'/de OR 'sex role'/de OR 'girl'/de OR 'gender identity'/de OR 'boy'/de OR 'decision making'/de OR 'self concept'/de) AND |
| Dynamic transmission model | ("ODE":ab,ti OR "differential equation*":ab,ti OR "ABM":ab,ti OR "agent based model*":ab,ti OR "mathematical model*":ab,ti OR "compartmental model*":ab,ti OR "SIR model*":ab,ti OR "susceptible infected recovered":ab,ti OR "SEIR model*":ab,ti OR "susceptible exposed infected recovered":ab,ti OR "susceptible infected susceptible":ab,ti OR "dynamic model*":ab,ti OR "dynamical model*":ab,ti OR "Markov model*":ab,ti OR "Markov chain":ab,ti OR "network analysis":ab,ti OR "network model*":ab,ti OR "system dynamic*":ab,ti OR "epidemic model*":ab,ti OR "pandemic model*":ab,ti OR "epidemiological model*":ab,ti OR "disease dynamic*":ab,ti OR "disease transmission*":ab,ti OR 'Markov chain'/exp OR 'epidemiological model'/exp OR 'mathematical model'/de OR 'compartment model'/de OR 'network analysis'/de OR 'prediction and forecasting'/de OR 'disease simulation'/de OR 'simulation'/de OR 'disease model'/de OR 'predictive model'/de OR 'communicable disease'/de) |

## Scopus

| **Concept** | **Search terms** |
| --- | --- |
| COVID-19, HIV, or malaria | (TITLE-ABS("COVID19") OR TITLE-ABS("COVID 19") OR TITLE-ABS("Coronavirus") OR TITLE-ABS("SARS-CoV-2") OR TITLE-ABS("Malaria*") OR TITLE-ABS("Plasmodium falciparum") OR TITLE-ABS("P. falciparum") OR TITLE-ABS("Plasmodium vivax") OR TITLE-ABS("P. vivax") OR TITLE-ABS("Plasmodium ovale") OR TITLE-ABS("P. ovale") OR TITLE-ABS("Plasmodium malariae") OR TITLE-ABS("P. malariae") OR TITLE-ABS("Plasmodium knowlesi") OR TITLE-ABS("P. knowlesi") OR TITLE-ABS("Human Immunodeficiency Virus") OR TITLE-ABS("HIV ") OR TITLE-ABS("HIV/AIDS")) AND |
| Sex/gender | (TITLE("women*") OR TITLE("girl*") OR TITLE("female*") OR TITLE("men") OR TITLE("men s") OR TITLE("boy s") OR TITLE("male*") OR TITLE-ABS("gender*") OR TITLE-ABS("sexbased") OR TITLE-ABS("sex based") OR TITLE-ABS("two spirit") OR TITLE-ABS("nonbinary") OR TITLE-ABS("non binary") OR TITLE-ABS("genderqueer") OR TITLE-ABS("genderfluid") OR TITLE-ABS("transgender*") OR TITLE-ABS("age structure") OR TITLE-ABS("age specific") OR TITLE-ABS("age distribution") OR TITLE-ABS("age stratif*") OR TITLE-ABS("stratified by age") OR TITLE-ABS("stratified on age") OR TITLE-ABS("age group*") OR TITLE-ABS("subpopulations by age") OR TITLE-ABS("decision making") OR TITLE-ABS("empowerment") OR TITLE-ABS("autonomy") OR TITLE-ABS("self efficacy") OR TITLE-ABS("transformative") OR TITLE-ABS("intersectionality")) AND |
| Dynamic transmission model | (TITLE-ABS("ODE") OR TITLE-ABS("differential equation*") OR TITLE-ABS("ABM") OR TITLE-ABS("agent based model*") OR TITLE-ABS("mathematical model*") OR TITLE-ABS("compartmental model*") OR TITLE-ABS("SIR model*") OR TITLE-ABS("susceptible infected recovered") OR TITLE-ABS("SEIR model*") OR TITLE-ABS("susceptible exposed infected recovered") OR TITLE-ABS("susceptible infected susceptible") OR TITLE-ABS("dynamic model*") OR TITLE-ABS("dynamical model*") OR TITLE-ABS("Markov model*") OR TITLE-ABS("Markov chain") OR TITLE-ABS("network analysis") OR TITLE-ABS("network model*") OR TITLE-ABS("system dynamic*") OR TITLE-ABS("epidemic model*") OR TITLE-ABS("pandemic model*") OR TITLE-ABS("epidemiological model*") OR TITLE-ABS("disease dynamic*") OR TITLE-ABS("disease transmission*")) |

## Global Health

| **Concept** | **Search terms** |
| --- | --- |
| COVID-19, HIV, or malaria | (("COVID19" OR "COVID 19" OR "Coronavirus" OR "SARS-CoV-2" OR "Malaria*" OR "Plasmodium falciparum" OR "P. falciparum" OR "Plasmodium vivax" OR "P. vivax" OR "Plasmodium ovale" OR "P. ovale" OR "Plasmodium malariae" OR "P. malariae" OR "Plasmodium knowlesi" OR "P. knowlesi" OR "Human Immunodeficiency Virus" OR "HIV " OR "HIV/AIDS").ti OR ("COVID19" OR "COVID 19" OR "Coronavirus" OR "SARS-CoV-2" OR "Malaria*" OR "Plasmodium falciparum" OR "P. falciparum" OR "Plasmodium vivax" OR "P. vivax" OR "Plasmodium ovale" OR "P. ovale" OR "Plasmodium malariae" OR "P. malariae" OR "Plasmodium knowlesi" OR "P. knowlesi" OR "Human Immunodeficiency Virus" OR "HIV " OR "HIV/AIDS").ab OR exp "coronavirus disease 2019"/ OR exp "Severe acute respiratory syndrome coronavirus 2"/ OR exp "malaria"/ OR exp "human immunodeficiency viruses"/) AND |
| Sex/gender | (("women*" OR "girl*" OR "female*" OR "men" OR "men s" OR "boy s" OR "male*" OR "gender*" OR "sexbased" OR "sex based" OR "two spirit" OR "nonbinary" OR "non binary" OR "genderqueer" OR "genderfluid" OR "transgender*" OR "age structure" OR "age specific" OR "age distribution" OR "age stratif*" OR "stratified by age" OR "stratified on age" OR "age group*" OR "subpopulations by age" OR "decision making" OR "empowerment" OR "autonomy" OR "self efficacy" OR "transformative" OR "intersectionality").ti OR ("gender*" OR "sexbased" OR "sex based" OR "two spirit" OR "nonbinary" OR "non binary" OR "genderqueer" OR "genderfluid" OR "transgender*" OR "age structure" OR "age specific" OR "age distribution" OR "age stratif*" OR "stratified by age" OR "stratified on age" OR "age group*" OR "subpopulations by age" OR "decision making" OR "empowerment" OR "autonomy" OR "self efficacy" OR "transformative" OR "intersectionality").ab OR exp "equal opportunities"/ OR exp "sexual roles"/ OR "gender relations"/ OR "sex differences"/ OR "boys"/ OR "girls"/ OR "females"/ OR "males"/ OR "self perception"/ OR "role perception"/ OR "women"/ OR "men"/) AND |
| Dynamic transmission model | (("ODE" OR "differential equation*" OR "ABM" OR "agent based model*" OR "mathematical model*" OR "compartmental model*" OR "SIR model*" OR "susceptible infected recovered" OR "SEIR model*" OR "susceptible exposed infected recovered" OR "susceptible infected susceptible" OR "dynamic model*" OR "dynamical model*" OR "Markov model*" OR "Markov chain" OR "network analysis" OR "network model*" OR "system dynamic*" OR "epidemic model*" OR "pandemic model*" OR "epidemiological model*" OR "disease dynamic*" OR "disease transmission*").ti OR ("ODE" OR "differential equation*" OR "ABM" OR "agent based model*" OR "mathematical model*" OR "compartmental model*" OR "SIR model*" OR "susceptible infected recovered" OR "SEIR model*" OR "susceptible exposed infected recovered" OR "susceptible infected susceptible" OR "dynamic model*" OR "dynamical model*" OR "Markov model*" OR "Markov chain" OR "network analysis" OR "network model*" OR "system dynamic*" OR "epidemic model*" OR "pandemic model*" OR "epidemiological model*" OR "disease dynamic*" OR "disease transmission*").ab OR exp "simulation models"/ OR "mathematical models"/ OR "dynamic models"/ OR "probabilistic models"/ OR "stochastic models"/ OR "disease models"/ OR "network analysis"/) |

# Methods for using ChatGPT for initial data extraction

We used ChatGPT for initial data extraction, with the exception of studies behind a paywall, using the following prompts. We explained the objective of the review and asked ChatGPT to make a spreadsheet with a defined set of columns from an uploaded PDF of an included study. We found that uploading one study at a time and having ChatGPT fill in the spreadsheet one row at a time produced more accurate results than analyzing multiple PDFs at once. After the first study was extracted using Prompt 1, we extracted data from the remaining studies using Prompt 2, one study at a time. ChatGPT did well extracting basic information (e.g., title, author, journal) but did not do well columns requiring more nuanced responses (e.g., explanation of disparities). We reviewed each cell in the spreadsheet created by ChatGPT manually and to make revision where necessary. The final spreadsheet is presented in Supplementary File 2 of included studies.

## Prompt 1

I am conducting a systematic scoping review of dynamic mathematical transmission models of COVID-19, HIV, and malaria. I need help extracting data from included studies. Attached is a study titled “Behaviour Change in Generalised HIV Epidemics”. Can you make a spreadsheet with the following columns using information from the uploaded study?

Columns:

1. Author: This is the last name of the first author followed by “et al.”. For example, “Hallett et al.”.
2. Title: The title of the study.
3. Year Published: The year the study was published.
4. Journal: The name of the journal that published the study.
5. Disease: This column lists the disease that the article focuses on. The choices are “COVID-19”, “HIV”, or “Malaria”.
6. Location of simulation: This column includes the location of the study population or what location the simulation is meant to represent.
7. Time period of simulation: This column includes the time period of the model simulation in "Month Year - Month Year" format. For example, if the study modeled the first wave of the COVID-19 pandemic from February 2020 to June 2020, this cell would state “Feb 2020 - Jun 2020”. If the month is not included in the study, just use “Year – Year” format.
8. Model Type: This column states the type of model used in the study. We are only considering dynamic models of infectious disease transmission, so your choices are: Compartmental, ABM, System Dynamics, DES, Markov. ABM stands for "agent-based model" and DES stands for "discrete event simulation". If you don’t think the study uses a dynamic model, state "NOT DYNAMIC?". You can include the name of the model in parentheses if it is well known or has been adapted from other studies. For example, “Compartmental (Asian Epidemic Model)”.
9. Data Sources: This is a list of data sources where the authors got the data to parameterize or validate their model. For example, “Population-based surveys, face-to-face interviews”.
10. Health Outcomes: This column includes the health outcomes modeled with the simulation. We are only interested in studies that model transmission of COVID-19, HIV, or Malaria. If you think the study is not a transmission model, please put "NOT TRANSMISSION MODEL?". If you do think the study is a transmission model, then we want to know the health outcomes modeled with the simulation. You're choices are: Infections, Deaths, Hospitalizations.
11. Other Outcomes: This column includes non-health outcomes modeled by the study. These may include "QALYs", "DALYs", or "ART coverage". If you think there are "Other outcomes" besides QALYs, DALYs, or ART coverage, please include them. For example, "Length of stay" or “Condom usage”.
12. Stratification (women): This column lists the subgroups of the population of women. How did the authors stratify the population of women (e.g., by age, sexual orientation, occupation, migrant status, injection drug use, refugee status, income, urbanicity (i.e., urban v rural). For example: “Brothel-based FSWs; Non-brothel-based FSWs; General women”.
13. Stratification (men): This column lists the subgroups of the population of men. How did the authors stratify the population of men (e.g., by age, sexual orientation, occupation, migrant status, injection drug use, refugee status, income, urbanicity (i.e., urban v rural).For example: “Heterosexual clients of FSWs; IDUs; MSM and transgender persons; General men”.
14. Stratification maintained: This column includes where in the article text, tables, or figures results are presented for the different gender groups. For example, if the study stratifies women by “general women” and “FSWs”, which table or figure shows results for these different subgroups. If you cannot find where the study includes these results, put "MAYBE IN SUPP?".
15. Explanation of why disparities exist: This column includes a short description (1-3 short sentences) of how the authors explain the presence of disparities in the context of this disease. For example, “Higher incidence among young women due cross-generational partnerships with HIV+ older men is presumed. Statistical analyses show reducing sex with older men and delaying sexual debut can reduce individual-level risk among young women.”
16. Scenarios modeled: This column lists the different scenarios or sensitivity analyses the authors tried when running their model. For example, “Reducing cross-generational sex, reducing age of sexual debut, and increasing condom use”.
17. Key findings: This column is a brief description of the key findings of the study with regards to the different gender-identity groups. For example, “Impact of behavioral change is sensitive to structure of network. Reducing cross-generational sex and age of sexual debut may have little population level impact unless risky behaviors are also reduced”.
18. Consideration of context in Discussion: This column gives 1-3 short sentences of how the authors put their study findings in context with their location of interest. For example, “Abstinence among young women has been credited with reducing HIV prevalence in Uganda, but population-level impact was likely attributed to increased condom use”.
19. Implications for decision making: This column includes a brief description of the implications for public health decision making that can be drawn from the article. For example, "Interventions should not only target particular at-risk groups but risky behavior throughout the sexual network”.

## Prompt 2

Great. Here is another study titled “Optimizing health resource allocation for improving timely HIV diagnosis in China”. Can you add a row to the spreadsheet with the relevant data from this study.
